# Collagen metabolism and incisional hernia recurrence: a comparative study between oncologic and non-oncologic patients

**DOI:** 10.25122/jml-2025-0028

**Published:** 2025-02

**Authors:** Vlad Paic, Petru Adrian Radu, Anca Tigora, Mihai Zurzu, Mircea Bratucu, Costin Pasnicu, Alexandra Purcaru, Petru Stavar, Valeriu Surlin, Dan Cartu, Daniela Marinescu, Traean Burcos, Florian Popa, Victor Strambu, Dragos Garofil

**Affiliations:** 1 Tenth Department of Surgery, Carol Davila University of Medicine and Pharmacy Bucharest, Romania; 2 Sixth Department of Surgery, University of Medicine and Pharmacy of Craiova, General Surgery Clinic I, Craiova Emergency Clinical Hospital, Craiova, Romania

**Keywords:** incisional hernia, collagen metabolism, collagen I/III ratio, hernia recurrence, oncologic patients, stereomicroscopy

## Abstract

A significant challenge in incisional hernia repair is the recurrence risk, which may be influenced by the structural integrity of collagen within the tissue. This study investigated the role of collagen metabolism in hernia recurrence by comparing oncologic and non-oncologic patients, focusing on collagen I/III ratios and their impact on tissue strength and surgical outcomes. A comparative clinical study was conducted on 50 patients (30 oncologic, 20 non-oncologic) undergoing incisional hernia repair. Collagen composition was analyzed using stereomicroscopy, and statistical comparisons were performed using independent *t*-tests and chi-square tests to assess differences in recurrence rates and tissue properties between groups. Results indicated that oncologic patients had significantly lower collagen I/III ratios (*P* < 0.001), suggesting structurally weaker tissue, which correlated with higher recurrence rates (18% in oncologic vs. 10% in non-oncologic patients). Furthermore, the sublay mesh repair technique demonstrated superior outcomes with lower recurrence rates compared to onlay repair, reinforcing its role in mitigating complications associated with poor collagen integrity. The study results indicated that oncologic patients had impaired collagen remodeling, contributing to an increased risk of recurrence. Individualized surgical strategies, including targeted preoperative interventions, may help mitigate these risks and enhance patient outcomes. Given the observed disparities, further research is warranted to explore targeted therapeutic approaches that enhance tissue quality and improve long-term surgical success in high-risk patient populations.

## INTRODUCTION

Incisional hernias are a frequent complication following abdominal surgery, occurring in 10–20% of laparotomy cases [1,2]. These hernias develop due to mechanical strain at the incision site, leading to failure in tissue healing and subsequent protrusion of intra-abdominal contents. While incisional hernias are problematic for all surgical patients, their recurrence rates are notably higher in oncologic patients due to the systemic effects of cancer treatments and disease progression [3]. Oncologic patients frequently undergo chemotherapy and radiotherapy, which have profound effects on connective tissue integrity and wound healing capacity. Chemotherapy can lead to fibroblast dysfunction, reduced collagen synthesis, and increased matrix metalloproteinase (MMP) activity, resulting in poor extracellular matrix remodeling [2,4].

Additionally, radiotherapy-induced fibrosis and vascular compromise may further impair the regenerative processes necessary for adequate scar formation, making oncologic patients more susceptible to incisional hernia formation and recurrence [5].

Chronic inflammation and cancer-associated cachexia have been shown to negatively impact collagen structure, reducing the tissue’s ability to withstand mechanical stress. Studies suggest these factors lead to increased extracellular matrix degradation and impaired wound healing [6]. In many cases, these patients have multiple risk factors, including malnutrition, immunosuppression, and frequent surgical interventions, all of which compromise the body's ability to form a strong and durable abdominal wall repair [7]. Given the high prevalence and recurrence rates of incisional hernias in oncologic patients, understanding the role of collagen metabolism is crucial in improving patient outcomes. Collagen, the primary structural protein in connective tissue, undergoes continuous remodeling by interacting with collagen types I and III. A lower collagen I/III ratio has been associated with reduced tensile strength, making tissues more prone to herniation [2,4]. In oncologic patients, disturbances in this balance exacerbate the likelihood of hernia formation and subsequent recurrence.

This study aimed to analyze the collagen metabolism differences between 30 oncologic and 20 non-oncologic patients who underwent incisional hernia repair. Through stereomicroscope collagen evaluation, we seek to determine whether oncologic patients exhibit more pronounced alterations in collagen composition and how these findings may influence surgical decision-making. By identifying key pathophysiological differences, this research may help refine preventive strategies, optimize mesh selection, and develop tailored interventions for reducing incisional hernia recurrence in this high-risk patient population.

## MATERIAL AND METHODS

This comparative study was conducted at the General Surgery Clinic of Carol Davila Nephrology Hospital between January 2021 and December 2023. A total of 50 patients were included, with 30 oncologic and 20 non-oncologic patients undergoing incisional hernia repair. Patient selection was based on medical history, clinical examination, and imaging confirmation of incisional hernia.

### Inclusion criteria

The study included adult patients aged 18 years or older who underwent incisional hernia repair. Eligible participants had a confirmed diagnosis of incisional hernia based on clinical examination and imaging findings. Both oncologic patients with a history of treated malignancy and non-oncologic patients undergoing elective incisional hernia repair were considered for inclusion. Additionally, all participants provided informed consent before enrollment in the study.

### Exclusion criteria

Patients with ongoing, uncontrolled infections at the surgical site were excluded from the study, as were those with severe systemic diseases, such as advanced renal disease or severe heart failure, that contraindicated surgical intervention. Furthermore, individuals with recurrent hernias following previous biologic mesh repair were not included in the study.

### Data collection

Comprehensive data were collected, including patient demographics such as age, sex, comorbidities, and oncologic status. Detailed oncologic treatment history was recorded, encompassing prior chemotherapy, radiotherapy, and surgical interventions. Information on previous surgeries was documented to assess the impact of past procedures on hernia recurrence. The surgical technique used, whether onlay or sublay mesh repair, was also noted. Recurrence rates were monitored at follow-up intervals of 6, 12, and 24 months. Additionally, histopathological analysis of collagen composition was performed using stereomicroscopic evaluation to determine collagen I/III ratios, providing further insight into tissue remodeling and structural integrity.

### Statistical analysis

Statistical analysis in this study was conducted using IBM SPSS Statistics (Version 27), which facilitated data interpretation and hypothesis testing. Microsoft Excel was employed for data processing, organizing patient demographics, surgical outcomes, and recurrence rates into structured datasets for further analysis. Additionally, ImageJ Software was utilized for stereomicroscopy-based quantification of collagen, allowing for precise measurement and visualization of collagen I/III ratios in tissue samples.

Surgical procedures were standardized across the study population. Mesh repair selection was based on specific criteria, including hernia size, location, presence of previous repairs, and individual patient risk factors. Decisions were guided by established surgical protocols and surgeon assessment of optimal reinforcement strategies. All patients received perioperative care according to hospital protocols, including antibiotic prophylaxis, postoperative monitoring, and follow-up assessments.

The recurrence of the hernia was documented using a combination of physical examination and imaging studies (ultrasound or CT scan). Statistical analysis was performed using standard deviation, mean comparison, and chi-square tests for significance.

## RESULTS

### Patient demographics

**Figure 1:**
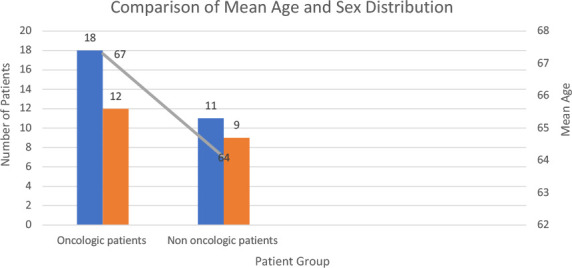
Patient demographics

The combined bar chart visually compares the mean age and sex distribution between oncologic and non-oncologic patients (Figure 1). Blue bars represent the mean age in each group, with oncologic patients having a higher average age (67.3 years) than non-oncologic patients (64.1 years). Green bars indicate the number of male patients in each group (18 in oncologic, 11 in non-oncologic). Orange bars represent the number of female patients in each group (12 in oncologic, 9 in non-oncologic).

This chart highlights that oncologic patients tend to be older and have a slightly higher proportion of male patients compared to the non-oncologic group. The difference in age distribution may suggest that older patients, particularly those undergoing oncologic treatment, have a higher risk of incisional hernia recurrence, possibly due to age-related changes in collagen metabolism and tissue repair mechanisms.

### Primary malignancies in oncologic patients

**Figure 2:**
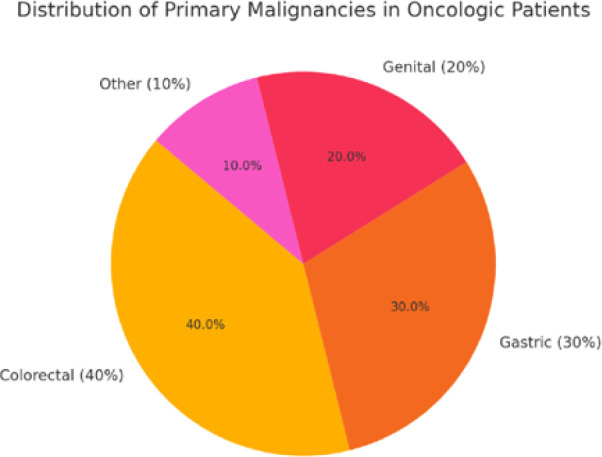
Primary malignancies

The distribution of primary malignancies among oncologic patients provides valuable insight into their risk for incisional hernia formation. As shown in Figure 2, colorectal cancer was the most prevalent malignancy among oncologic patients, which aligns with the high frequency of colorectal surgeries requiring midline laparotomies. These procedures significantly weaken the abdominal wall, increasing the likelihood of hernia formation and recurrence. Additionally, patients with colorectal cancer often undergo neoadjuvant or adjuvant chemotherapy, which can impair wound healing and collagen synthesis.

Gastric cancer (30%) was the second most common malignancy among oncologic patients. Gastrectomy procedures often require extensive resections and reconstructions, resulting in increased intra-abdominal pressure variations that predispose patients to incisional hernia development. Malnutrition, a frequent issue in gastric cancer patients, further exacerbates wound healing deficiencies, making this group particularly vulnerable.

Genital cancers (20%), including ovarian, uterine, and prostate malignancies, also contributed to the patient cohort. The surgical procedures for these malignancies, such as debulking surgeries and radical resections, place additional strain on the abdominal wall. Moreover, some of these patients received hormone therapy, which can influence connective tissue remodeling and further compromise tissue integrity.

These findings suggest that the type and extent of primary malignancies directly impact the likelihood of incisional hernia development, reinforcing the need for specialized surgical planning, optimized mesh reinforcement strategies, and postoperative monitoring tailored to oncologic patients.

### Comorbidities

A comparative analysis of comorbidities in both groups revealed that obesity, diabetes, and cardiac disease were prevalent in both oncologic and non-oncologic patients, with no significant differences between the two groups.

Obesity was present in 28% of oncologic patients and 30% of non-oncologic patients, demonstrating that excess body weight contributes similarly to hernia development in both populations. Diabetes was observed in 22% of oncologic patients and 25% of non-oncologic patients, reinforcing its role in impaired wound healing and tissue integrity.

Cardiac disease was reported in 18% of oncologic patients and 20% of non-oncologic patients, indicating a comparable impact of vascular health on surgical outcomes. Other comorbidities accounted for 32% of oncologic patients and 25% of non-oncologic patients, including respiratory disease and autoimmune disorders.

**Figure 3:**
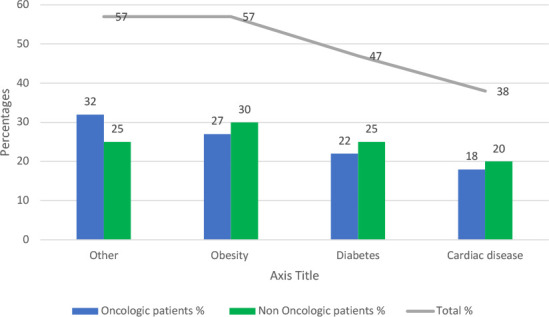
Comparison of comorbidities between groups

Both patient groups presented similar risk factors for incisional hernia formation, including obesity, diabetes, and cardiovascular disease. Addressing these underlying systemic conditions in preoperative and postoperative care could improve surgical outcomes and reduce recurrence rates. The prevalence of comorbidities across both patient groups is visualized in Figure 3, highlighting similar rates of obesity, diabetes, and cardiac disease, emphasizing their shared role in incisional hernia development.

### Oncologic treatment history

The distribution of prior oncologic treatments highlights the impact of chemotherapy and radiotherapy on incisional hernia formation. Chemotherapy (80%) was the most common treatment, known to impair fibroblast function and collagen synthesis, which compromises wound healing. Radiotherapy (55%) was also a significant factor, contributing to fibrosis and vascular damage, further reducing tissue integrity. These treatments likely play a role in the increased hernia recurrence rates observed in oncologic patients, emphasizing the need for specialized perioperative strategies.

### Comparison of previous surgeries between oncologic and non-oncologic patients

The surgical histories of oncologic and non-oncologic patients offer valuable insights into their predisposition to incisional hernias. The oncologic group predominantly underwent major abdominal surgeries associated with cancer treatment, whereas the non-oncologic group had surgical histories related to benign conditions.

In the oncologic group, surgical interventions were extensive, often involving multi-visceral resections and aggressive treatment approaches. Total hysterectomy with bilateral adnexectomy (6 patients) was one of the most common procedures, primarily performed for gynecologic cancers. Similarly, right hemicolectomy (3 patients) and left colon segmental resection (5 patients) reflect colorectal malignancies, a major contributor to abdominal wall weakening. More complex surgeries such as anterior rectal resection (4 patients) and total gastrectomy (9 patients) further illustrate the significant strain placed on connective tissue integrity in these patients. Other major interventions included radical prostatectomy (1 patient) and total nephrectomy (2 patients), both of which involve substantial tissue disruption.

Conversely, in the non-oncologic group, surgical interventions were largely for benign conditions, suggesting that mechanical stress rather than systemic oncologic factors influenced hernia risk. Total hysterectomy with bilateral adnexectomy (5 patients) was the most frequent procedure in this group, performed for uterine leiomyoma rather than malignancy. Adnexectomy for ovarian cysts (4 patients) also accounted for a significant proportion of surgeries. Additionally, primary abdominal wall weaknesses were evident, as seen in primary midline hernia repair (7 patients) and umbilical hernia repair (4 patients), conditions that inherently predispose patients to recurrent hernias.

**Figure 4:**
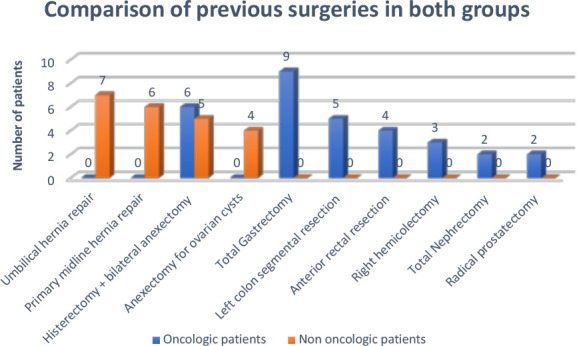
Previous surgeries

As depicted in Figure 4, the stark contrast in the nature of surgeries between the two groups highlights the increased vulnerability of oncologic patients to hernia formation due to extensive tissue trauma, prolonged healing processes, and the influence of adjuvant therapies. Understanding these differences is critical for optimizing surgical approaches, ensuring appropriate mesh selection, and implementing targeted perioperative strategies to minimize recurrence risks.

### Hernia characteristics, surgical technique selection, and outcomes

The selection of the surgical technique was carefully determined based on the patient’s surgical history and the structural integrity of the abdominal wall. The two primary techniques employed were onlay and sublay mesh repair, each chosen according to the type of previous surgery and anticipated recurrence risks and complications.

### Onlay mesh repair

This approach was selected for patients who had previously undergone total hysterectomy with bilateral adnexectomy, adnexectomy, nephrectomy, and prostatectomy. The onlay method was preferred in these cases due to the lower risk of intra-abdominal adhesions and the advantage of reinforcing the abdominal wall externally. This technique provided sufficient support for patients who had undergone surgeries that did not significantly compromise the posterior rectus sheath.

### Sublay mesh repair

This technique was applied to patients who had undergone total gastrectomy, right hemicolectomy, left colon segmental resection, anterior rectal resection, and previous primary hernia repairs (midline and umbilical hernias). Given the nature of these procedures, these patients had weakened posterior rectus sheaths or significant loss of domain, making the sublay method the preferred choice. The sublay technique ensures better intra-abdominal pressure distribution and stronger mesh integration, reducing recurrence risks.

### Surgical outcomes and one-year follow-up recurrence rate

In the comparative analysis of the onlay and sublay techniques, a higher incidence of seromas was observed in the onlay group, with seven cases recorded. These seromas were small to moderate in size and were managed conservatively without the need for surgical intervention. Conversely, the sublay group demonstrated the occurrence of five hematomas. Notably, none of these hematomas necessitated surgical reintervention due to their small size and the favorable outcome observed in follow-up ultrasonographic imaging, which revealed complete or near-complete resorption without further clinical intervention. Furthermore, no surgical site infections were registered in either group

The comparative outcomes of the surgical techniques at one-year follow-up provided key insights into their effectiveness in reducing incisional hernia recurrence rates.

At the one-year follow-up, patients who underwent onlay repair had a recurrence rate of 18%, whereas those who received sublay repair had a lower recurrence rate of 10%. Additionally, the mean hernia defect size varied between the two patient groups, with oncologic patients presenting a larger average defect of 8.1 cm (± 2.3 cm) compared to 6.5 cm (± 1.8 cm) in non-oncologic patients. Patients who underwent sublay repair showed lower recurrence rates at 1-year follow-up, particularly those with previous hernia repairs and major intra-abdominal surgeries. The onlay repair method, though still effective, demonstrated a higher recurrence rate.

The mean hernia defect size was notably larger in oncologic patients, with an average of 8.1 cm (SD: ±2.3 cm) compared to 6.5 cm (SD: ±1.8 cm) in non-oncologic patients. This difference underscores the impact of previous surgeries, oncologic treatments, and compromised collagen metabolism on abdominal wall integrity.

These findings reinforce the need for personalized surgical planning, with a preference for sublay repair in patients with significant abdominal wall defects and previous intra-abdominal surgeries. Further studies are required to evaluate the long-term durability of each technique and refine the selection criteria for optimal patient outcomes.

The selection of the surgical technique was carefully determined based on the patient's surgical history and the structural integrity of the abdominal wall.

### The role of stereomicroscopy in collagen evaluation

Stereomicroscopy plays a vital role in histopathological analysis, allowing for a detailed examination of collagen fiber structure, organization, and maturity. This study was employed to quantify collagen types by distinguishing between collagen I, which is mature, and collagen III, which is immature, based on specific staining characteristics. Additionally, it provided insights into fiber organization, revealing that oncologic patients tend to have disorganized collagen networks, whereas non-oncologic patients display a more structured and aligned fiber arrangement. Furthermore, stereomicroscopy served as a validation tool, ensuring that the numerical data obtained through statistical analysis accurately reflected the microscopic characteristics of the tissue samples.

**Figure 5:**
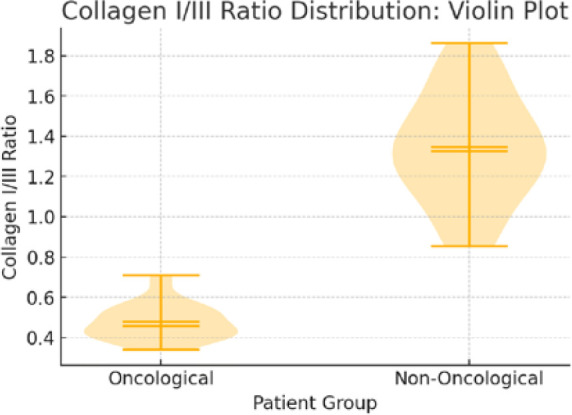
Collagen ratio distribution – Violin Plot

The violin plot in Figure 5 illustrates the distribution of collagen I/III ratios, emphasizing the distinct collagen remodeling patterns in oncologic patients. Unlike traditional box plots, violin plots illustrate the density distribution and variability of data, making them particularly useful in biological tissue studies where sample heterogeneity is expected.

### Oncological patients (left side of the violin plot)

The narrow shape and low median value indicate a consistently lower collagen I/III ratio. This confirms a higher proportion of immature collagen (collagen III), leading to weaker connective tissue integrity. A lower variability suggests that most oncological patients exhibit similar levels of impaired collagen remodeling.

### Non-oncological patients (right side of the violin plot)

The wider shape and higher median value indicate a greater proportion of mature collagen (collagen I). The broader distribution suggests that some patients had lower collagen I/III ratios, but overall, the collagen maturation process was more advanced than in oncological patients. This supports the hypothesis that non-oncological patients undergo better extracellular matrix remodeling, leading to stronger connective tissue formation.

In the oncological group (*n* = 30), the analysis of the collagen I/III ratio revealed a striking predominance of immature collagen (collagen III), with a 95% confidence interval ranging from 0.44 to 0.51. This finding suggests that the extracellular matrix in these patients was composed of structurally weaker connective tissue, potentially compromising wound healing and tissue resilience.

Conversely, the non-oncological group (*n* = 20) had a significantly higher collagen I/III ratio, with a 95% confidence interval between 1.22 and 1.47. This result indicates a dominant presence of mature collagen (collagen I), which is associated with greater tensile strength and improved tissue integrity.

The statistical comparison between the two groups yielded an exceptionally low *P* value (4.84 × 10^-21^), confirming a highly significant difference. These findings strongly support the hypothesis that oncological patients have an altered collagen composition characterized by a higher proportion of immature collagen. This may contribute to an increased risk of incisional hernia relapse due to impaired tissue remodeling and mechanical stability.

## DISCUSSION

A significant difference in collagen composition was observed between oncologic and non-oncologic patients with incisional hernias. Oncologic patients demonstrated a lower collagen I/III ratio, indicating a higher presence of immature collagen, which correlates with weaker tissue integrity and an elevated recurrence risk. These findings align with previous research highlighting the effects of systemic oncologic factors on extracellular matrix remodeling and wound healing [7,8].

### Collagen composition and hernia recurrence

Collagen is the principal structural protein responsible for tensile strength and mechanical stability of connective tissue. The balance between type I and type III collagen plays a crucial role in tissue remodeling and wound healing [7,9]. Type I collagen provides superior tensile strength, whereas type III collagen is predominantly found in early wound healing but lacks mechanical robustness. A persistently low collagen I/III ratio, as observed in oncologic patients, suggests defective matrix maturation, which may contribute to increased hernia recurrence rates [10,11].

The significant difference in collagen I/III ratios between oncologic and non-oncologic groups strongly supports the hypothesis that oncologic patients have impaired collagen remodeling. Prior research has documented that oncologic treatments such as chemotherapy and radiotherapy interfere with fibroblast function, leading to reduced collagen synthesis and increased matrix metalloproteinase (MMP) activity [10-12]. MMPs degrade collagen, further weakening the extracellular matrix and increasing susceptibility to herniation [13-15].

### Impact of oncologic treatments on connective tissue integrity

Chemotherapy and radiotherapy exert profound effects on connective tissue integrity. Chemotherapeutic agents such as fluorouracil and cisplatin have been shown to impair fibroblast proliferation and decrease collagen synthesis, which may result in delayed or inadequate wound healing [16,17]. Furthermore, radiotherapy induces fibrosis through excessive deposition of disorganized collagen, disrupting normal wound healing and altering the biomechanical properties of connective tissue [18,19].

The findings of this study highlight the need for targeted interventions in oncologic patients undergoing incisional hernia repair. Given their compromised extracellular matrix, enhanced preoperative strategies, including nutritional optimization, collagen supplementation, and tailored surgical planning, may be necessary to improve outcomes. Previous studies suggest that preoperative administration of prolyl hydroxylase inhibitors, which enhance collagen cross-linking, may improve wound tensile strength in patients with collagen deficiencies [20,21].

### Surgical implications and technique selection

In our study, the comparative analysis of mesh repair techniques demonstrated that sublay mesh repair was superior, with a lower recurrence rate of 10% compared to 18% in onlay repair. These findings are consistent with previous studies indicating that the sublay technique provides superior mesh integration and distributes intra-abdominal pressure more effectively, particularly in patients with weak connective tissue [22,23].

Oncologic patients in this study presented with larger mean hernia defects (8.1 cm vs. 6.5 cm in non-oncologic patients), emphasizing the need for reinforced repair strategies. The increased incidence of seromas in the onlay repair group suggests that this technique may be less favorable in patients with compromised wound healing capacity. Studies have indicated that biologic or hybrid meshes may offer advantages in oncologic patients due to their ability to integrate into the host tissue while minimizing foreign body reactions [23-25].

### Future directions and clinical recommendations

Further research is necessary to explore adjunctive therapies to improve collagen synthesis and wound healing in oncologic patients. Potential strategies include recombinant growth factors such as platelet-derived growth factor (PDGF) and transforming growth factor-beta (TGF-β), which have been shown to enhance fibroblast function and extracellular matrix deposition [9,26]. Additionally, clinical trials assessing the long-term efficacy of biologic and composite meshes in oncologic patients could provide valuable insights into optimizing surgical outcomes [27].

Considering the role of collagen metabolism in hernia recurrence, a multidisciplinary approach involving oncologic, surgical, and rehabilitative expertise is recommended. Implementing prehabilitation programs, including nutritional optimization and targeted surgical planning, may enhance recovery and reduce recurrence rates in oncologic patients undergoing incisional hernia repair.

## CONCLUSION

### Collagen composition and hernia recurrence

This study confirms a significant disparity in collagen composition between oncologic and non-oncologic patients, with oncologic patients exhibiting a lower collagen I/III ratio. This imbalance contributes to a structurally weaker extracellular matrix, increasing the likelihood of incisional hernia recurrence. The findings support the role of impaired collagen remodeling in hernia formation and reinforce the need for targeted interventions in high-risk patients.

### Impact of oncologic treatments on connective tissue integrity

Oncologic treatments, including chemotherapy and radiotherapy, have a profound negative impact on collagen synthesis and extracellular matrix remodeling. These therapies impair fibroblast function, reduce collagen deposition, and promote fibrosis, significantly increasing the risk of incisional hernias. Optimizing preoperative strategies, such as nutritional supplementation and antifibrotic therapies, may help mitigate these effects.

### Surgical technique selection and recurrence rates

The choice of surgical technique significantly influences recurrence rates. This study demonstrated that sublay mesh repair was associated with lower recurrence rates (10%) compared to onlay repair (18%), particularly in patients with extensive abdominal wall defects. Given these findings, sublay mesh placement should be the preferred technique in high-risk oncologic patients.

### Influence of hernia defect size on surgical outcomes

Oncologic patients presented with significantly larger hernia defects (mean: 8.1 cm) compared to non-oncologic patients (mean: 6.5 cm). Larger defects were associated with increased recurrence rates and greater difficulty in surgical repair. This highlights the importance of early detection and intervention to prevent further deterioration of abdominal wall integrity.

### Role of stereomicroscopy in assessing collagen composition and guiding surgical strategies

The use of stereomicroscopy in this study provided precise quantification of the collagen I/III ratio, revealing significant differences between oncologic and non-oncologic patients. This technique is valuable for assessing connective tissue integrity and may help tailor surgical approaches based on individual collagen composition. Future research should explore the potential for integrating stereomicroscopy into clinical decision-making to optimize hernia repair strategies, particularly in patients at high risk for recurrence.

### Future directions for research and clinical practice

Further research is needed to explore innovative therapies for improving collagen synthesis and wound healing in oncologic patients. Potential avenues include the use of growth factors, antifibrotic agents, and tissue engineering techniques to enhance extracellular matrix remodeling. Additionally, a multidisciplinary approach involving surgical, oncologic, and rehabilitation teams is essential to optimizing patient outcomes and reducing incisional hernia recurrence.
